# The impact of probiotic administration on experimental *in vitro* and *in vivo* infection by *Trypanosoma cruzi*

**DOI:** 10.1590/0074-02760240272

**Published:** 2025-10-20

**Authors:** Denise da Gama Jaén Batista, Lara Calheiros Missagia, Kelly Cristina Demarque, Gabriel Melo de Oliveira, Marcos Meuser Batista, Maria de Nazaré Correia Soeiro

**Affiliations:** 1 Fundação Oswaldo Cruz-Fiocruz, Instituto Oswaldo Cruz, Laboratório de Biologia Celular, Rio de Janeiro, RJ, Brasil

**Keywords:** Lactobacillus rhamnosus, multi strain PB8, Trypanosoma cruzi, experimental chemotherapy, benznidazole

## Abstract

**BACKGROUND:**

Chagas disease (CD) caused by *Trypanosoma cruzi* has limited therapy. Probiotics sustain healthy microbiota, playing roles in biological events.

**OBJECTIVES:**

Our aim was to determine the impact of probiotics on *T. cruzi* infection *in vitro* and in mouse acute experimental models.

**METHODS:**

The multi strain - PB8 and the *Lactobacillus rhamnosus* (LR) were orally administered [10^6^-10^9^ colony-forming units (CFU)] for seven days prior to mice infection followed for 14 daily administrations. Peritoneal mouse macrophages (PMM) were obtained from mice treated with 10^9^ probiotics one day before collection and infected *in vitro* with or not benznidazole (BZ).

**FINDINGS:**

LR and PB8 reduced by 44-87% and 23-16% the parasitaemia peak in male and female mice, respectively, but did not protect against mortality. Histopathology showed mild reduction in cardiac nests due to probiotics’ administration. PB8 and LR suppressed the parasite infection of PMM by 24 and 26%, reaching 65 and 42% of declines, respectively when 3% thioglycolate was performed. PB8 increased BZ activity at 1 µM, reaching 40% of parasitism’ declines compared to BZ alone (25%). No gender difference was noticed during probiotic *in vivo* administration.

**MAIN CONCLUSIONS:**

The results point to the potential of a combined therapeutic approach for CD, using probiotics and BZ.

Chagas disease (CD) is caused by the protozoan parasite *Trypanosoma cruzi*. CD is currently endemic in 21 Latin American countries with more than 6 million people infected, and around 10,000 deaths per year.^1^ However, it is still commonly associated with poor and rural regions.^2^ The principal route of *T. cruzi* infection is via infected faeces of haematophagous triatomine bugs,^2^ ^,^ ^3^ although transmission can also occur orally, via the placental route during pregnancy or childbirth; by blood transfusion and organ transplantation; or even, from laboratory accidents.^4^

The infection period and the symptoms may vary depending on the route of penetration, the inoculum, the strain, and the patient’s condition.^5^ ^,^ ^6^ The disease has two consecutive clinical steps: a short acute phase followed by the chronic stage.^7^ The acute phase is characterised by detectable parasitaemia, but most carriers are asymptomatic, and less than 5% die due to encephalomyelitis or severe heart failure and due to cardiac arrest.^7^ ^,^ ^1^

Due to immune and inflammatory responses, parasitism is controlled, but not eliminated, and the infected individuals migrate to the chronic phase. Most will remain asymptomatic throughout their lives, in the clinical stage called the indeterminate form of the disease. However, after years, 30-40% may develop symptoms related to cardiac and/or digestive disorders, leading to severe loss of the organs functionally.^7^

Currently, the therapy is based on two old medications, nifurtimox (Nif, LAMPIT^®^, Bayer) and benznidazole (BZ/LAFEPE, Abarax^®^, ELEA and BZ/Chemo Research, Exeltis).^8^ Both achieve satisfactory results during the acute phase but require prolonged use and induce side effects that result in abandonment of treatment. Besides, both drugs present lower cure rates in the later chronic phase.^9^ ^,^ ^10^

The microbiota is composed of diverse microorganisms, including commensals, symbiotics and even pathogenic microbes.^11^ In humans, microbiota acts on different metabolic pathways including nutritional metabolism, immune response, drug interactions besides impacting disease outcomes.^11^ ^,^ ^12^ ^,^ ^13^ Its high diversity and density are influenced by different environmental, physiological, and behavioural factors such as habits and food sources, gender, age, physical activity, among others.^14^ ^,^ ^15^

Thus, microbial communities can also influence the susceptibility or resistance profile to an infectious agent.^16^ For example, diseases caused by protozoa and helminths can be aggravated or their pathological consequences reduced according to the intestinal microbiota.^11^ ^,^ ^17^ Experimental data showed that it may influence and provide protection against infections caused by bacteria spp. such as *Listeria monocytogenes*, *Salmonella typhimurium*, *Salmonella enteritidis* and *Escherichia coli*,^18^ by virus such as coronavirus^19^ and by protozoan parasites like *T. cruzi*.^20^ ^,^ ^21^ It has been reported that mouse experimental infection by *T. cruzi* is more severe in germ-free animals, resulting in higher parasitaemia and greater mortality.^20^ Also, the oral or intraperitoneal administration of *Lactobacillus casei* to female Swiss mice decreases *T. cruzi* parasitaemia.^21^

Probiotics are live microorganisms that may promote healthy microbiota, maintaining a balance among the different axis (intestinal, skin, oral and genital mucosa) and promoting health benefits for the host.^11^ Thus, it has been suggested that these elements could contribute as co-therapies against parasitic infections, being a low-cost and non-invasive therapeutic approach, not inducing adverse effects.^22^

In the present study, we investigated through *in vitro* and *in vivo* assays the possible impact of probiotics administration during murine experimental acute *T. cruzi* infection and its potential effect during the therapy with BZ.

## MATERIALS AND METHODS

*Probiotics and reference drug* - A multi strain probiotic (eight strains composed of: *Lactobacillus acidophilus LA-14*, *Bifidobacterium lactis BL-04*, *Lactobacillus plantarum LP-115*, *Lactobacillus salivarius LS-33*, *Bifidobacterium bifidum BB-06*, *Bifidobacterium longum*, *Lactobacillus rhamnosus* GG (DSM 33156) - LGG^*®*^ and *Lactobacillus casei LC-1110*) were acquired from Nutrition Now and Hypera and diluted in phosphate buffered saline (PBS). The *Lactobacillus rhamnosus* GG (DSM 33156) - LGG^*®*^ was purchased by Mantecorp Farmasa. BZ, the reference drug for CD, was purchased from LAFEPE.

*Animals* - Male and female Swiss mice (18 - 21 g) were obtained from the Institute of Science and Technology in Biomodels (ICTB-Fiocruz), housed in a maximum of six animals per box and maintained at 20 to 24ºC under a cycle light/dark 12/12 h. Animals were kept in a specific pathogen-free (SPF) and received sterilised water and food ad libitum [ration Nuvilab, Quimtia composed by corn, soybean meal, wheat bran, calcium carbonate, dicalcium phosphate, sodium chloride (common salt), vitamin A, vitamin D3, vitamin E, vitamin K3, vitamin B1, vitamin B2, vitamin B6, vitamin B12, niacin, calcium pantothenate, folic acid, biotin, choline chloride, iron sulphate, manganese monoxide, zinc oxide, copper sulphate, calcium iodate, sodium selenite, cobalt sulphate, lysine and methionine] . The animals were acclimatised for seven days before the experiments.

*Parasites* - Bloodstream trypomastigote (BT) forms of *T. cruzi* [Y strain - discrete typing units II (DTU II)] were obtained from the blood of *T. cruzi*-infected mice at the peak of parasitaemia using 3.8% of sodium citrate as anticoagulant.^23^

*Mammalian cells* - Peritoneal mouse macrophages (PMM) were obtained from Male Swiss mice previously stimulated or not with 3% thioglycolate, 96 h before PMM obtention.^24^ Briefly, mice stimulated or not by thioglycolate, were treated orally with probiotics (10^9^) 24 h before the obtention of PMM. Briefly, 10 mL of RPMI medium was injected into the peritoneum of the euthanised animal. The peritoneal cells were subsequently collected using a syringe, the suspension quantified in a Neubauer chamber for adjusting the cellular density to be seeded in 96-well plates (10^5^ cells per well) or 24 wells plates (3x 10^5^ cells). After 2 h of plating, fresh RPMI medium supplemented with 10% foetal bovine serum (FBS), 2%. L-glutamine and 1% penicillin and streptomycin were added and the PMM incubated at 37ºC (5% CO_2_).^24^

*In vitro activity on intracellular forms of T. cruzi (Y strain) in PMM obtained from animals stimulated or not with 3% thioglycolate and treated with probiotics* - PMM from mice stimulated or not with thioglycolate and treated or not for 24 h with 10^9^ LR and PB8 were obtained, seeded as above described and infected for another 24 h at 37ºC with BT of *T. cruzi* (Y strain, under parasite: host cell ratio 10:1). After the infection of cells, the cultures were washed to remove parasites that did not internalise and incubated or not for 48-96 h with different concentrations (1 and 10 µM) of the antiparasitic compound. Alternatively, PMM were incubated *in vitro* for 2 and 24 h with probiotics, rinsed with PBS and subsequently infected for 24 h with BT forms (ratio of 10 parasites to 1 cell). The cultures were rinsed with PBS, fixed with Bouin, stained with Giemsa.^25^ Subsequently, the percentage of infected host cells, as well as the number of parasites per cell were quantified under a light microscope to determine the infection index, which represents the multiplication of these two factors.^25^

All assays were performed in duplicates at least three times.

*In vivo assays*: *infection and treatment* - Male and female Swiss mice were treated by gavage for seven consecutive days with 10^6^ - 10^9^ colony-forming units (CFU) of *Lactobacillus rhamnosus* (LR) and PB8 (10^9^ CFU). After treatment, mice were infected intraperitoneally (i.p.) with 10^4^ BT forms (Y strain) of *T. cruzi*. Then, probiotic administration was sustained for another 14 days, totalising 21 days of treatment. In all assays, control groups included a group infected and treated only with vehicle and a group infected and treated with the reference drug (BZ, at optimal dose - 100 mg/kg/day, given by gavage at 5-14-day post-infection - dpi).

*Parasitaemia, weight curve and mortality rate* - Parasitaemia was measured according to the Pizzi-Brener methodology (Brener, 1962). From the 5th (parasitaemia onset) up to 16 dpi, parasitaemia analysis was performed individually by light microscopy observation of the number of parasites in 5 μL of blood collected from the tail vein^26^ Mortality was assessed daily and expressed as a percentage of accumulated mortality.^26^ ^,^ ^27^

*Histopathological analysis* - At the 10 dpi, the surviving animals were euthanised using an overdose of isoflurane and confirmed by the absence of respiratory movement (apnoea), absence of heartbeat (asystole), loss of colour of the mucous membranes, and loss of brightness and moisture of the corneas, as observed by the veterinarian responsible for the procedure. Then, the heart collected and fixed in formalin (10%).^26^ The samples were cleaved and processed for paraffin inclusion. The obtained sections (7 µm) were then stained using haematoxylin and eosin dyes, as reported.^26^ The number of amastigote nests in at least 50 fields (100 x magnification in light microscope) per slide from ≥ 3 mice per group (four sections from each animal) was quantified. Inflammation was assessed regarding the presence of: (i) only inflammatory infiltrates classified as “mononuclear infiltrate”; and (ii) cell infiltrates associated to other tissular alterations such as degeneration, congestion, and necrosis, classified as “mononuclear inflammation”. The degree of the tissular lesions was determined based on the extent and severity of inflammation was classified scoring grades 0-3: (a) Grade 0: minimal or no tissular alterations and the lack of infiltration or inflammation; (b) Grade 1: mild tissular changes, with the presence of infiltration or inflammation restricted to small areas (only in the atrial cardiac region and focal, multifocal or diffuse); (c) Grade 2: moderate tissular changes with infiltration and/or inflammation covering most of the atrial and part of the ventricular heart regions in a multifocal pattern and multifocal and (d) Grade 3: severe tissular changes, with infiltration and/or inflammation largely found in the samples covering most of the atrial and ventricular cardiac region in a multifocal or diffuse manner.

*Statistical analysis* - The analysis was performed using Analysis of variance (ANOVA) with a significance level of p ≤ 0.05 [95% confidence interval (CI)].

*Ethical statement* - All procedures were carried out under the biosafety guidelines of the Oswaldo Cruz Institute (IOC/ Fiocruz), and all animal procedures complied with the guidelines established by the FIOCRUZ Committee of Ethics for the Use of Animals, resolution (CEUA numbers: L038-2017 and L-038/2017-A4).

## RESULTS

**Fig. 1:**
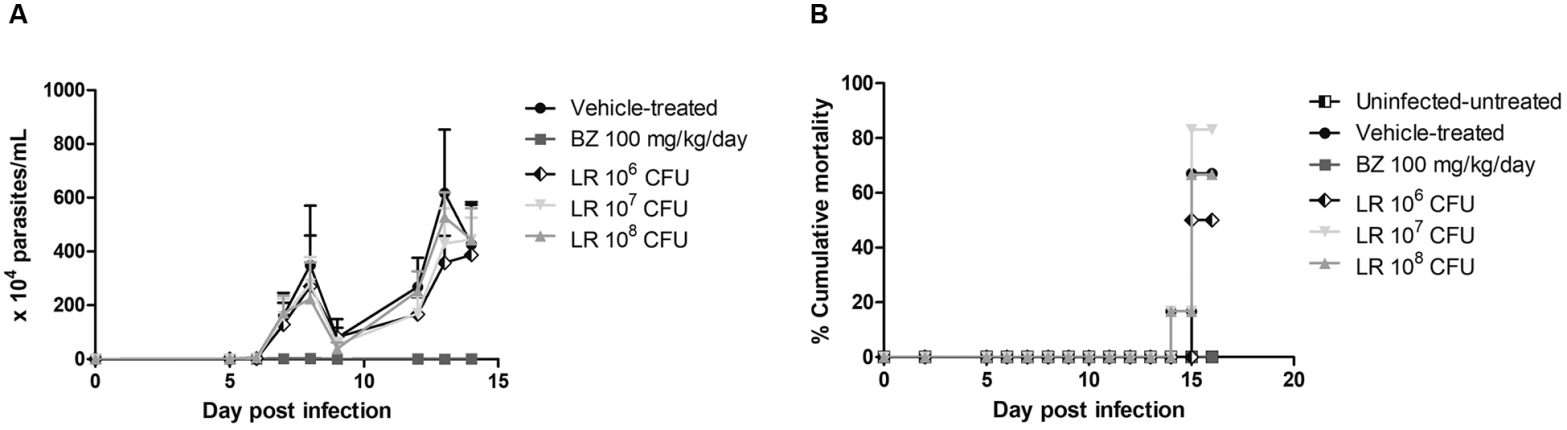
the effect of 10^6^-10^8^ colony-forming units (CFU) *Lactobacillus rhamnosus* (LR) in Swiss male mice before experimentally infection by *Trypanosoma cruzi* (Y strain). The graph depicts the parasitaemia curve (A) and cumulative mortality (B) (n = 5). In all groups treated with probiotics, there was no statistical significance (p > 0.05) as compared to the reference group (vehicle-treated animals).

The administration of increasing CFU (10^6^ - 10^8^) of LR showed decreases (no statistical significance - p > 0.05) in the parasitaemia peak (at 8 dpi) of Swiss male mice acutely infected with *T. cruzi*, reaching 36% at 10^8^ CFU. As positive control, the treatment of the infected animals for five days with the reference drug (BZ at 100 mg/kg/day) cleared the parasitaemia (Fig. 1A). Regarding mortality rates, only BZ gave 100% animal survival (Fig. 1B).

**Fig. 2:**
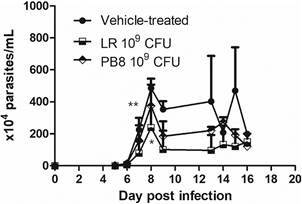
the effect of 10^9^ colony-forming units (CFU) *Lactobacillus rhamnosus* (LR) and PB8 oral administration in Swiss male mice before experimentally infection by *Trypanosoma cruzi* (Y strain). The graph depicts the parasitaemia curve (A) and the parasite load at the parasitaemia peak (8 dpi) (B) (n = 5). Statistical analysis compared to the reference group (vehicle-treated animals): *p = 0.039 and **p = 0.25.

Swiss male mice treated with higher probiotic concentration (LR 10^9^ CFU) showed a significant decrease (51%, p = 0.039) in the mean number of circulating parasites in the peak (8 dpi) as compared to the vehicle group (Fig. 2A). Importantly, two out of five animals treated with probiotics showed negative parasitaemia at the 8 dpi (Fig. 2B). A head-to-head comparison using a multi strain probiotic (PB8 - 10^9^ CFU) also demonstrated parasitaemia decline (23%, p = 0.25), being less expressive than LR (Fig. 2A).

**Fig. 3:**
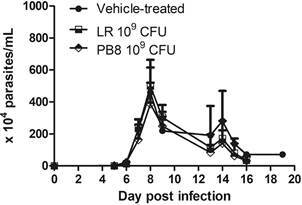
the effect of 10^9^ colony-forming units (CFU) *Lactobacillus rhamnosus* (LR) and PB8 on the parasitaemia peak (8 dpi) in Swiss female mice experimentally infected by *Trypanosoma cruzi* (Y strain). The vehicle group received only saline before infection (n = 5). In all groups treated with probiotics, there was no statistical significance (p > 0.05) as compared to the reference group (vehicle-treated animals).

In the female Swiss mice group only PB8 (10^9^ CFU) mildly reduced (p = 0.44) the parasitaemia peak by 15.92% (Fig. 3).

**Fig. 4:**
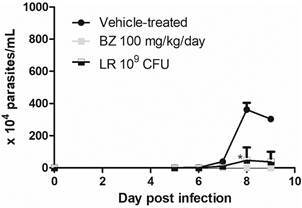
the effect of 10^9^ colony-forming units (CFU) *Lactobacillus rhamnosus* (LR) oral administration in Swiss male mice before the experimental infection by *Trypanosoma cruzi* (Y strain). The graph denotes the parasitaemia curve over the days of the experiment. Statistical analysis as compared to the reference group (vehicle-treated animals): *p = 0.038.

**Fig. 5:**
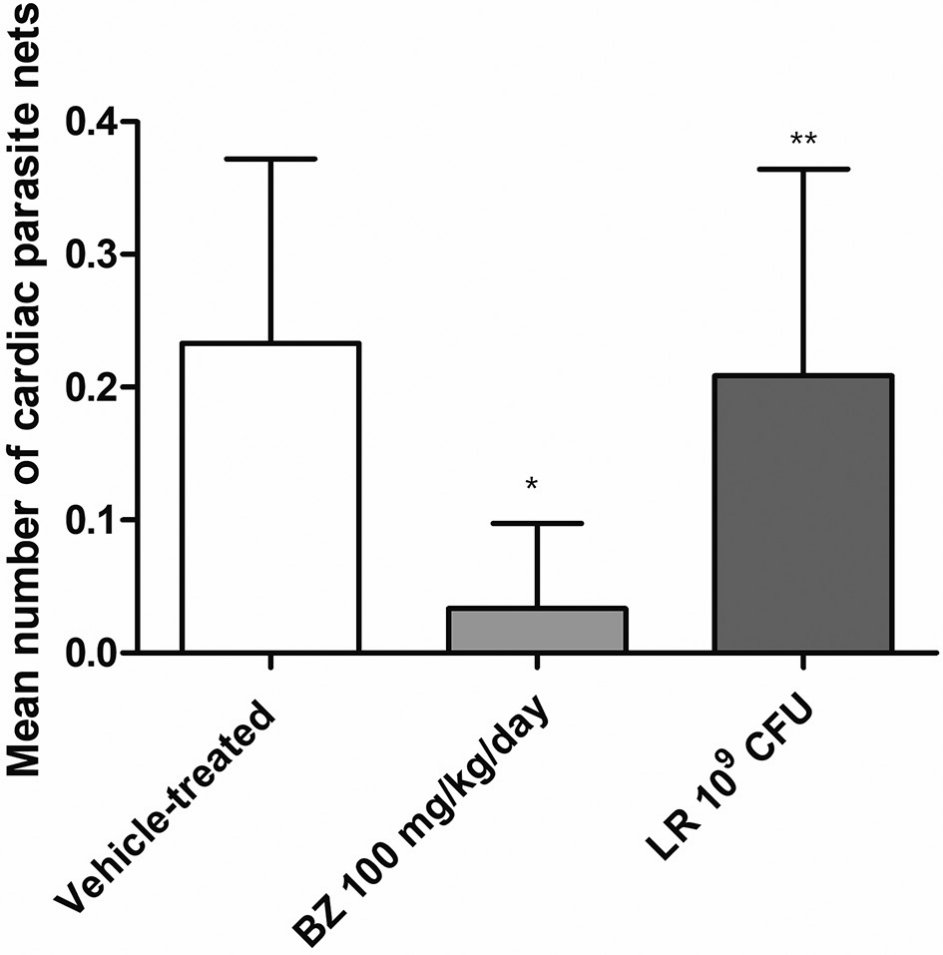
the effect on cardiac parasitism of Swiss male mice experimentally infected by *Trypanosoma cruzi* (Y strain). The mean number of parasite nests in cardiac samples of Swiss male mice submitted to 10^9^ colony-forming units (CFU) *Lactobacillus rhamnosus* (LR) administration and then experimentally infected with *T. cruzi*. In parallel, *T. cruzi*-infected animals were treated for five consecutive days with benznidazole (BZ) (100 mg/kg/day) (n = 3). Statistical analysis as compared to the reference group (vehicle-treated animals): *p = 0.0069 and **p = 0.74.

As LR was more effective than PB8, a further assay was performed to compare the probiotic activity at parasitaemia as well as in the heart parasitism. Swiss male mice treated with LR achieved 87% (p = 0.038) of parasitaemia decline at the 8 dpi (Fig. 4). The heart histopathology at the 10 dpi demonstrated a 12% (p = 0.74) decrease in the number of cardiac nests as compared to vehicle group, while BZ group reached 85% (p = 0.0069) (Fig. 5). The analysis of cardiac inflammatory profile demonstrated that only animals treated with BZ were scored as “grade 1” (inflammatory infiltrate). Animals that received LR (except one scored as grade 1) showed cardiac mononuclear inflammation. Vehicle-treated animals showed both grades 1 and 2 (Table).

**TABLE:** Histopathological analysis of heart samples from male mice treated with probiotic or benznidazole (BZ) treatment. The table depicts the degree of inflammation and infiltration profile in the heart of the studied animals’ groups

Group	Inflammatory profile^*^ /number of animals			
	0	1	2	3
Vehicle	0/3	2/3	1/3	0/3
BZ	1/3	2/3	0/3	0/3
LR 10^9^	0/2	1/2	0/2	1/2

LR: *Lactobacillus rhamnosus*. *Inflammatory profile scored as: (a) Grade 0: minimal or no tissular alterations and the lack of infiltration or inflammation; (b) Grade 1: mild tissular changes, with the presence of infiltration or inflammation restricted to small areas (only in the atrial cardiac region and focal, multifocal or diffuse without significant mucosal involvement in intestinal samples); (c) Grade 2: moderate tissular changes with infiltration and/or inflammation covering most of the atrial and part of the ventricular heart regions in a multifocal pattern and multifocal and/or diffuse infiltration/inflammation without significant mucosal intestinal involvement; and (d) Grade 3: severe tissular changes, with infiltration and/or inflammation largely found in the samples covering most of the atrial and ventricular cardiac region in a multifocal or diffuse manner and multifocal or diffuse involving all layers of the intestine.

**Fig. 6:**
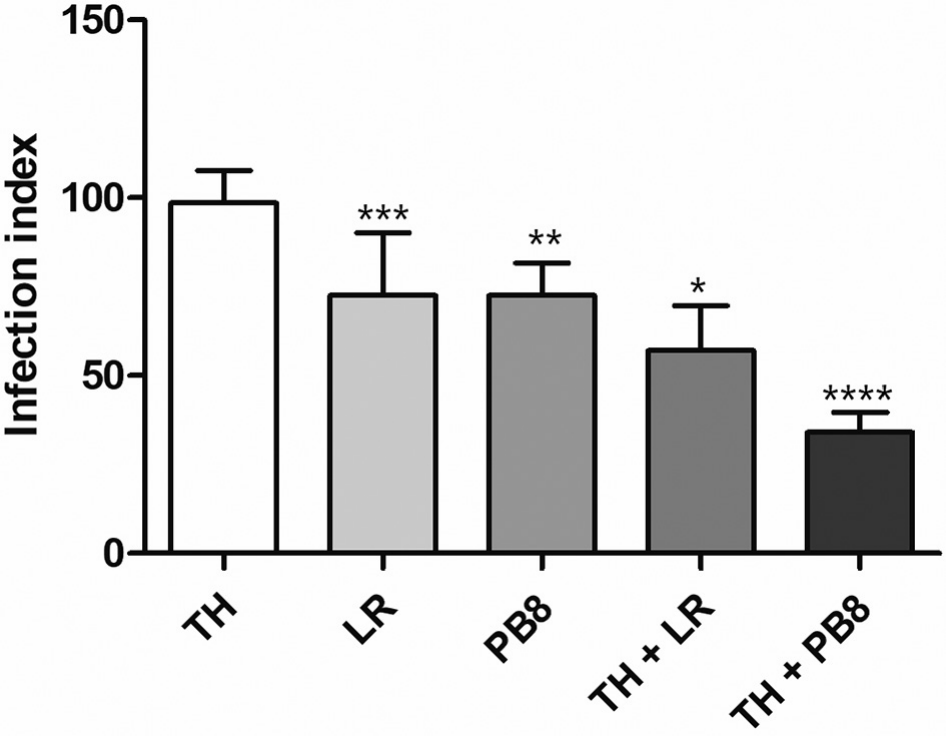
the effect of the infection indexes of peritoneal mouse macrophages (PMM) infected by *Trypanosoma cruzi* (Y strain) *in vitro* collected from male Swiss mice previously exposed to a single dose of 10^9^ colony-forming units (CFU) *Lactobacillus rhamnosus* (LR) and PB8. The graph shows the infection rate of PMM from animals also stimulated or not only with 3% thioglycolate (TH) and treated or not with the probiotics (n = 3). Statistical analysis as compared to the reference group (TH-treated animals): ****p = 0.01, **p = 0.11 and ***p = 0.2; and *p = 0.06.

Our *in vivo* findings encouraged us to further explore if the probiotic administration could impact the *in vitro* infection of PMM by *T. cruzi*. Our findings demonstrate that a single *in vivo* administration of PB8 and LR (without thioglycolate stimulation *in vivo*) followed by collection and infection of PMM with *T. cruzi* reduced the infection index by 24 and 26% (p = 0.11 and p = 0.2), respectively as compared to the administration of only TH. This decline was more evident when animals were both stimulated using thioglycolate and probiotics. The association of probiotics administration *in vivo* with thioglycolate stimulation reached drops of 65 and 42% for PB8 (p = 0.01) and LR (p = 0.06), respectively, as compared to PMM obtained only with thioglycolate stimulation (Fig. 6).

**Fig. 7:**
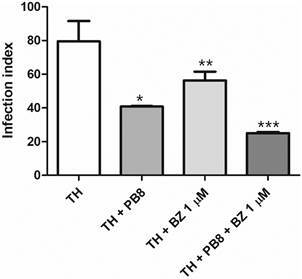
the effect of the infection index of peritoneal mouse macrophage (PMM) infected with *Trypanosoma cruzi* (Y strain) *in vitro* under incubation of benznidazole (BZ). PMM were obtained from thioglycolate (TH) stimulated animals submitted or not to 10^9^ colony-forming units (CFU) PB8 administration, infected with *T. cruzi* (Y strain) and further exposed or not for 48 h to BZ at 1 µM (n = 3). Statistical analysis: *TH x TH/PB8 (p = 0.044); **TH x TH/BZ (p = 0.129) and ***TH/PB8 x TH/PB8/BZ (p = 0.0012).

Next, we checked if the probiotic administration could also impact on the antiparasitic effect of the reference drug used to treat CD. We confirmed reductions in the infection index of PMM when cells were collected from male mice exposed to PB8+TH as compared to only TH stimulation (50% decline, p = 0.044; Fig. 7).

When a high concentration of BZ (10 µM) was used to treat the infected-PMM cultures, no difference was achieved (data not shown). However, when a suboptimal concentration (1 µM) was assessed, a clear improvement in BZ activity was found. BZ alone at 1 µM reduced 25% (p = 0.129) the intracellular parasitism of PMM from mice stimulated with thioglycolate. PMM from PB8-treated mice stimulated by thioglycolate exposed to 1 µM BZ (TH/PB8 + BZ), displayed a statistically significant drop of 40% (p = 0.0012) in the intracellular parasitism as compared to TH/PB8 group (Fig. 7).

## DISCUSSION

A large bulk of evidence suggests that microbiota plays important roles on host health, improving immunological and metabolic functions, demonstrating the potential use of probiotics. Clinical and preclinical studies report the benefits of probiotic administration as adjuvant therapy for many diseases including neuropsychiatric disorders,^28^ infectious diseases^21^ among others.^29^ Presently, we addressed if probiotic administration in experimental mice could improve their response to control a parasitic infection caused by the intracellular protozoan *T. cruzi*.

Two types of probiotics were tested: a multi strain probiotic composed of eight different strains (*Lactobacillus acidophilus LA-14*, *Bifidobacterium lactis BL-04*, *Lactobacillus plantarum LP-115, Lactobacillus salivarius LS-33, Bifidobacterium bifidum BB-06*, *Bifidobacterium longum*, *Lactobacillus rhamnosus* and *Lactobacillus casei LC-1110*) comparing to the effect of a single strain (*LR*) present into the multi strain probiotic. Our data showed the benefit of probiotic use as adjuvant, achieving reductions in blood and cardiac parasitism of mice experimentally infected by *T. cruzi*. Our results support and corroborate the findings reported by Martins and colleagues using another *T. cruzi* strain and the administration of *L. casei*.^30^

Our assays showed that the probiotic effect was more pronounced in male mice as compared to females. Differences related to parasitic infection and therapy susceptibility were already reported while comparing both genders during experimental mouse models of *T. cruzi* infection,^27^ but further studies are needed as only single analysis was performed using female Swiss mice.

The drop in the parasitaemia peak observed during probiotic administration was confirmed by the histopathological analysis of cardiac samples of the surviving animals, although in a less extensive manner. This analysis validates the hypothesis that healthy microbiome helps to sustain an effective immunological response, contributing to parasitism control.

Our histopathologic analysis showed an inflammatory profile in the hearts of all animals regardless of the therapy that is due to the direct action of the parasite during the acute infection,^31^ associated or not with degeneration, congestion, necrosis, and/or fibrosis.^32^

The bulk of our data supports the use of probiotics as an additive element along with antiparasitic drugs. These may be different mechanisms such as substances released by probiotic microorganisms, as well as their action in the inflammatory and immune responses.^33^ Literature reports on modulation of the cellular microenvironment (nutrients, mucus, receptor availability on epithelial cells, pH, tight junctions, and peristalsis, among others), production of biologically active molecules (such as bacteriocins, antibiotics, or hydrogen peroxide) with antimicrobial properties. Also, immunological modulation has been claimed due to the interaction of phagocytes such as macrophages and dendritic cells and T cells, leading to cytokine induction and/or a humoral immune response.^34^

We also found that animals treated with probiotic (one single administration of 10^9^ CFU of PB8 and LR) triggered the capacity of PMM to control *in vitro* infection by *T. cruzi*, resulting in lower infection rates. This effect was even more pronounced when mice were additionally stimulated with thioglycolate, a drug used for triggering macrophages migration into the mice peritoneum. We found that more than 60% of decreases in the parasitism rates of PMM obtained from PB8+TH stimulated mice. Also, when the reference drug for CD was added at 10 µM into the infected PMM cultures, no additional effect could be observed possibly because this concentration already corresponds to the IC_90_ value of BZ.^8^ However, when a ten-fold lower concentration (1 µM) was used, a statistically significant decline in the infection index was observed: BZ alone resulted in 25% of decreases at 1 µM, while PMM collected from probiotic-treated and/or not TH stimulated mice and further exposed to BZ achieved > 40% of decline in the infection index.

Our results unequivocally show that probiotics can promote the action of antiparasitic agents at suboptimal doses justifying additional studies.
